# Harnessing inter-disciplinary collaboration to improve emergency care in low- and middle-income countries (LMICs): results of research prioritisation setting exercise

**DOI:** 10.1186/s12873-020-00362-7

**Published:** 2020-08-31

**Authors:** Fiona E. Lecky, Teri Reynolds, Olubukola Otesile, Sara Hollis, Janette Turner, Gordon Fuller, Ian Sammy, Jean Williams-Johnson, Heike Geduld, Andrea G. Tenner, Simone French, Ishtar Govia, Julie Balen, Steve Goodacre, Sujan B. Marahatta, Shaheem DeVries, Hendry R. Sawe, Mohamed El-Shinawi, Juma Mfinanga, Andrés M. Rubiano, Henda Chebbi, Sang Do Shin, Jose Maria E. Ferrer, Mashyaneh Haddadi, Tsion Firew, Kathryn Taubert, Andrew Lee, Pauline Convocar, Sabariah Jamaluddin, Shahzmah Kotecha, Emad Abu Yaqeen, Katie Wells, Lee Wallis

**Affiliations:** 1 School of Health and Related Research, University of Sheffield, Sheffield, and Emergency Deparment, Salford Royal Hospital, Salford, UK; 2 World Health Organisation, Geneva, Switzerland; 3 Scarborough General Hospital, Tobago, Canada; 4 The University of West Indies, Kingston, Jamaica; 5 Divsion of Emergency Medicine, Stellenbosch University, Cape Town, South Africa; 6 University of California San Francisco, San Francisco, USA; 7 Manmohan Memorial Institute of Health Sciences, Kathmandu, Nepal; 8 Emergency Medical Services for the Western Cape Government, Cape Town, South Africa; 9 Emergency Medical Association of Tanzania (EMAT), Dar es Salaam, Tanzania; 10 Muhimbili University of Health and Allied Science, Dar es Salaam, Tanzania; 11 Ain Shams University, Cairo, Egypt; 12 Muhimbili National Hospital, Dar es Salaam, Tanzania; 13 Neurosciences Institute, El Bosque University, Bogotá, Colombia; 14 Colombian Trauma Association, Bogotá, Colombia; 15 Ministry of Health, Bab Saadoun, Tunisia; 16 Seoul National University Hospital, Seoul, South Korea; 17 American Heart Association (AHA), Dallas, USA; 18 Ministry of Health, Tehran, Iran; 19 Columbia University, Emergency Medicine, New York, NY USA; 20 Ministry of Health, Addis Ababa, Ethiopia; 21 American Heart Association (AHA), Geneva, Switzerland; 22 Philippine College of Emergency Medicine, Parañaque, Philippines; 23 Sungai Buloh Hospital, Sungai Buloh, Malaysia; 24 Bugando Medical Centre, Mwanza, Tanzania; 25 Ministry of Health, Amman, Jordan; 26 Divsion of Emergency Medicine, University of Vermont, Burlington, Vermont USA; 27 Division of Emergency Medicine, University of Cape Town, F51 Old Main Building, Groote Schuur Hospital Observatory, Cape Town, South Africa

**Keywords:** Global Health, Research prioritisation, Quality indicators, Emergency care systems, Low resource settings

## Abstract

**Background:**

More than half of deaths in low- and middle-income countries (LMICs) result from conditions that could be treated with emergency care - an integral component of universal health coverage (UHC) - through timely access to lifesaving interventions.

**Methods:**

The World Health Organization (WHO) aims to extend UHC to a further 1 billion people by 2023, yet evidence supporting improved emergency care coverage is lacking. In this article, we explore four phases of a research prioritisation setting (RPS) exercise conducted by researchers and stakeholders from South Africa, Egypt, Nepal, Jamaica, Tanzania, Trinidad and Tobago, Tunisia, Colombia, Ethiopia, Iran, Jordan, Malaysia, South Korea and Phillipines, USA and UK as a key step in gathering evidence required by policy makers and practitioners for the strengthening of emergency care systems in limited-resource settings.

**Results:**

The RPS proposed seven priority research questions addressing: identification of context-relevant emergency care indicators, barriers to effective emergency care; accuracy and impact of triage tools; potential quality improvement via registries; characteristics of people seeking emergency care; best practices for staff training and retention; and cost effectiveness of critical care – all within LMICs.

**Conclusions:**

Convened by WHO and facilitated by the University of Sheffield, the Global Emergency Care Research Network project (GEM-CARN) brought together a coalition of 16 countries to identify research priorities for strengthening emergency care in LMICs. Our article further assesses the quality of the RPS exercise and reviews the current evidence supporting the identified priorities.

## Abreviations

ECSAs Emergency Care System Assessments

ECS Emergency Care Systems

GETI Global Emergency and Trauma Care Initiative

GEM-CARN Global Emergency Care Research Network project

HICs High-income countries

JLA James Lind Alliance

LMICs ow- and middle-income countries

RPS research prioritisation setting

RPS research priority setting

RCEM Royal College of Emergency Medicine

WHO The World Health Organization

UHC Universal health coverage

## Introduction

While prevention is ideal, there is no context in which all emergencies can be averted, and prevention strategies may take years or decades to show benefit [1, 2]. Globally 90% of healthcare emergencies occur in low- and middle income countries (LMICs) [3, 4] especially in children and working age adults [5]. The World Bank Disease Control Priorities Project estimates that over half of deaths in LMICs result from conditions that could be treated with emergency care [6]. Emergency care is an essential component of universal health coverage (UHC) and serves as the first point of contact with the health system for many. However, the majority of people around the world remain without timely access to high-quality essential emergency care services, and this results in enormous disparities in outcomes [7]. People with similar injuries, for example, are nearly twice as likely to die in LMICs than in high-income countries (HICs) [8]. In HICs, Emergency Care Systems (ECS) have evolved considerably over the last 50 years alongside the development of Emergency Medicine as a distinct medical specialty, recognising the need for training, expertise and dedicated systems to care effectively for the acutely ill and injured of all ages [9]. Recent studies point to the benefits of utilising research evidence to reconfigure ECS elements in HICs [10]. As an example, a 19% reduction in risk adjusted mortality following serious injury has been observed following the introduction of major trauma centres and management networks in the UK [11].

Studies such as this one highlight the importance of defining research priorities to inform strengthening of Emergency Care Systems, but there is little research to guide policy and implementation in settings where resources are limited and prioritization is critical. World Health Assembly Resolution 72.16 calls for national-level WHO Emergency Care System Assessments (ECSAs) to define system-level gaps and priorities for action and highlights the need for a stronger evidence base to inform policy and implementation [12]. While there have been prior emergency care research priority setting (RPS) exercises oriented to the global context, these have largely focused on general frameworks or on logistical and ethical challenges of conducting emergency care research in LMICs [13–15], or on consensus-based prioritisation of quality indicators for emergency care provision in LMICs [16], rather than identifying specific research questions. One 2013 initiative identified potential priority research questions, though without the benefit of input from policymakers and implementers [17, 18]. Other efforts have been limited to HIC settings: the Royal College of Emergency Medicine (RCEM) collaborated with the James Lind Alliance (JLA) to engage clinicians, patients, carers and the public to prioritise the top ten research questions in the UK Emergency Medicine [19]. A wide variety of research priority setting (RPS) exercises have been undertaken by WHO in the areas of infectious and communicable disease [20]. The recent launch of the Global Emergency and Trauma Care Initiative (GETI) [21] will facilitate scale-up and roll-out of the WHO ECS Toolkit, including coordinated implementation and concentrated monitoring across countries in all WHO regions. However, WHO has not yet undertaken this process for pathways to care for people with life-threatening and/or time-sensitive conditions.

**Fig. 1:**
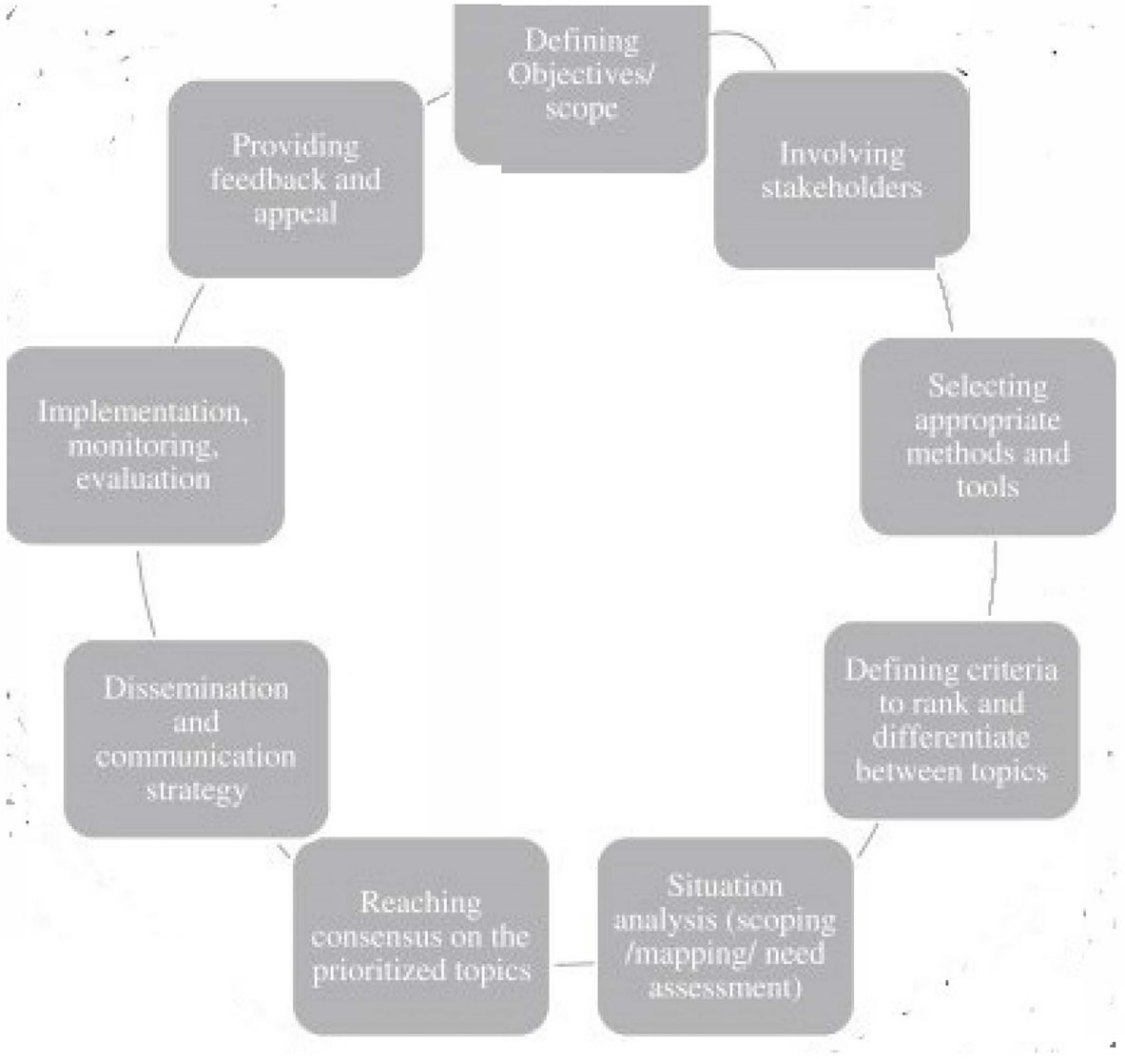
Wheel of Research Priority Setting Exercises (This figure was published in the Journal of Clinical Epidemiology,Volume number 66(5), Nasser M, Ueffing E, Welch V, Tugwell P, An equity lens can ensure an equity-oriented approach to agenda setting and priority setting of Cochrane Reviews, Pages 511–521. Copyright© Elsevier Inc. 2013)

The Global Emergency Care Research Network project (GEM-CARN), an international and multidisciplinary coalition of researchers and stakeholders across different countries and regions, conducted a RPS exercise after the 2019 WHO Global Emergency Care Systems meeting to identify evidence gaps and emergency care research priorities in LMICs. In this article, we explore four phases of research prioritisation, identify seven priorities for improving emergency care systems in LMICs, assess the quality of our RPS exercise and review the current evidence available. Our RPS process was informed by using steps identified during a Cochrane International workshop on Research Priority Setting Methods [22] (see Fig. 1), published evidence reviews, consensus documents and gap analyses from country experts [5, 7, 23] as well as early data from WHO emergency care implementation activities using the WHO Emergency Care Toolkit, which includes national system-level assessments, clinical and process guidance for emergency units [24]. Details of WHO Tools and other materials used to inform the RPS process are provided in Table 1.

**Table 1:** Documents informing phase 1 of research prioritisation setting exercise

Document	
WHO Emergency Care System Framework [25]	Highlights the essential components of an emergency care system.
Emergency Care System assessment [26]	A process executed at the national level in which countries bring together key stakeholders to undertake a structured appraisal of the essential system components needed to deliver care for emergency conditions, including injury. Each element of the emergency care system (as visualized in the Emergency Care System Framework mentioned above) is assessed. ECSA results are used to develop country roadmaps and implementation plans.
WHO-ICRC Basic Emergency Care course [26]	Targeted at frontline prehospital personnel and linked with the WHO *Emergency Triage Assessment and Treatment* for children, and the *IMAI Quick Check and Emergency Treatments* for adults.
WHO Trauma Care Checklist [26]	Guides clinical teams through basic critical steps of trauma care.
Key Systematic reviews [5, 23]	• Obermeyer et al. Emergency care in 59 low- and middle-income countries: a systematic review. Bull World Health Organ 2015; 93:577-586G • Kironji et al. Identifying barriers for out of hospital emergency care in low and low-middle income countries: a systematic review. BMC Health Services Research 2018; 18: 291
AFEM proceedings [16]	Broccoli et al. Defining quality indicators for emergency care delivery: findings of an expert consensus process by emergency care practitioners in Africa. BMJ Global Health 2018; 3:e000479.

## Method

### Phase 1: engaging with stakeholders, identifying questions and uncertainties

**Fig. 2:**
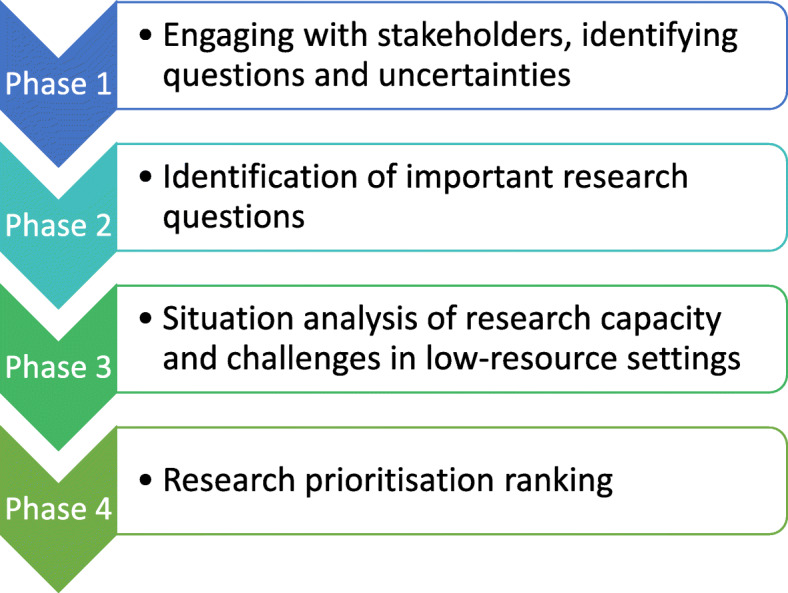
Four phases of Global Emergency Care Research Network Research Prioritisation Setting Exercise

**Fig. 3:**
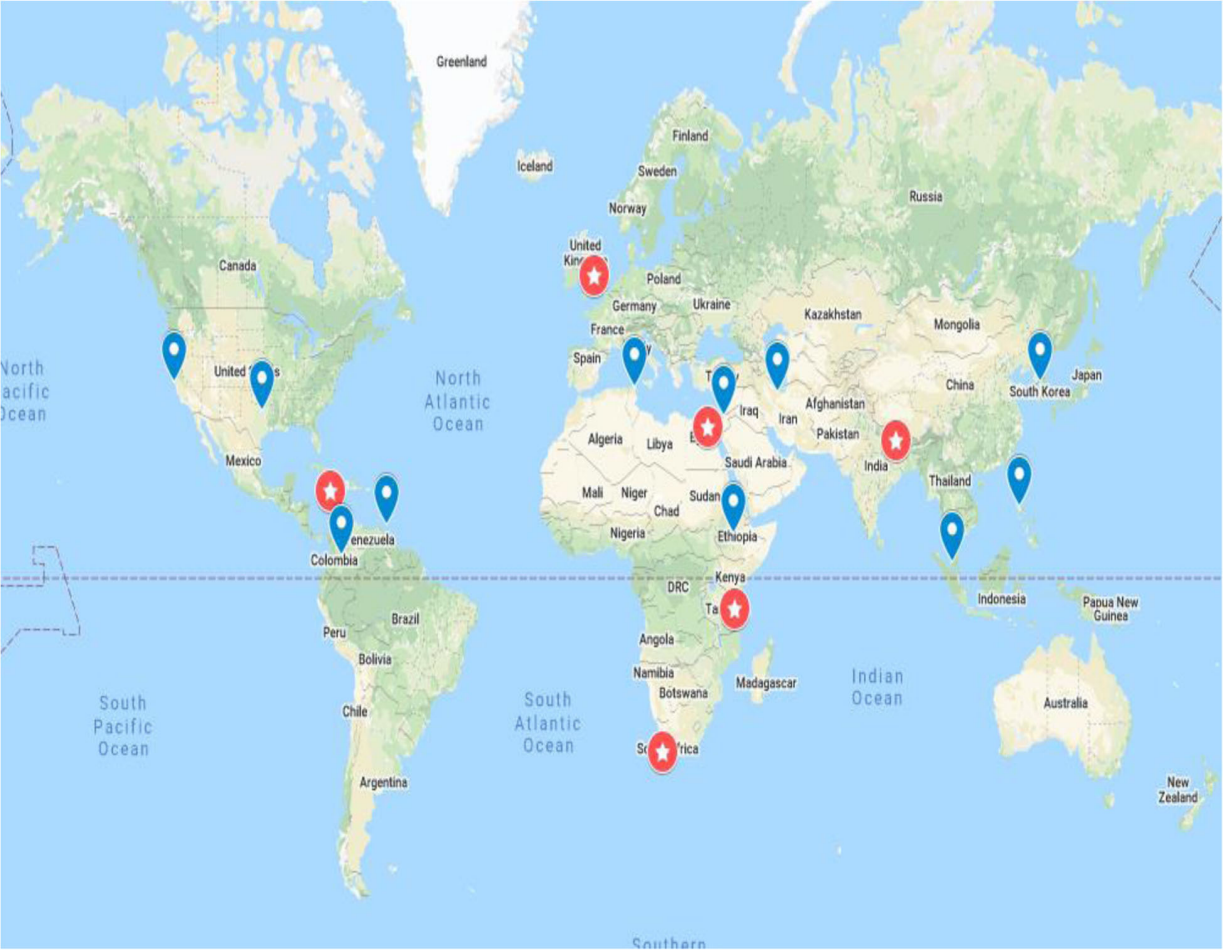
Sixteen countries participating in Global Emergency Care Research Network (GEMCARN) Research Priority Setting Exercise (Highlighted Red = GEMCARN partners, Blue = GEMCARN collaborators) Map taken from copyright free image https://www.sheffield.ac.uk/library/copyright/imagesource, country locators added with photoshop

Engaging with stakeholders was the first phase of the RPS exercise Fig. 2. The 2019 WHO Global ECS meeting in Geneva, Switzerland in February 2019 presented an opportunity to convene six universities of the initial GEM-CARN project group (The University of Cape Town, South Africa; Ain Shams University, Cairo, Egypt; Manmohan Memorial Institute of Health Sciences, Nepal; University of the West Indies, Jamaica; Muhimbili University of Health and Allied Sciences, Tanzania; The University of Sheffield) with representatives from ten additional countries, bringing the total number of countries represented to 16. The convening brought together researchers from relevant disciplines including Emergency Medicine, Pre-hospital Care, Health Services Research, Public Health, Disaster Management and Defence (Military) Medicine. Beyond the GEM-CARN universities, the group included representatives from: Ministry of Health Tunisia; Philippine College of Emergency Medicine, Philippines; American Heart Association (AHA), USA; Ministry of Health Ethiopia; Scarborough General Hospital, Tobago; Ministry of Health, Iran; Sungai Buloh Hospital, Malaysia; Seoul National University Hospital South Korea; Ministry of Health, Jordan; Colombian Trauma Association, Colombia; and the University of California, San Francisco WHO Collaborating Centre for Emergency and Trauma Care, USA Fig. 3. Collaborators engaged with stakeholders to discuss current emergency care delivery across national contexts and to identify current gaps in the evidence for the effectiveness of emergency care interventions.

An overview of the *WHO Emergency Care System Framework* was presented [25]. The ensuing round table discussion highlighted significant evidence gaps to support Emergency Care System (ECS) development in most LMICs. The LMICs represented highlighted different stages of ECS development across and within countries- particularly with regards to prehospital care. The discussion also reflected a concentration of emergency medicine and supporting specialty expertise in university teaching hospital emergency departments/Facilities and the impact of “(lack of) ability to pay” and other non-clinical factors that impacted on patient access to ECS. It was felt that collaborative interdisciplinary research holds the potential to deliver better understanding of this ECS heterogeneity and its impact across countries. The discussion identified potential areas of interest for future research studies, including evaluating the impact of national system-level assessments on country planning and implementation. In addition, it was felt that research studies focused on understanding and developing context-relevant standards and measurement priorities for emergency care across countries were important if the effects of change and impact are to be reliably measured. Furthermore, there is a need to understand the case mix in LMICs better by matching resources with case mix such as non-communicable and end-stage diseases. The discussion reflected on the challenges of sustainable measurement of emergency care quality indicators within a limited-resource system. It was also noted that research initiatives should take account of feasibility, the need for open-access platforms, and development of low cost continuous feedback and benchmarking systems.

### Phase 2: identification of important research questions

In phase 2, participants were asked to identify important research questions for improving the effective delivery of emergency care in low-resource settings. Using modified nominal group techniques [27, 28], participants were divided into three groups to brainstorm potential research questions that should be prioritized for improving emergency care in low-resource settings. Each group had a mix of LMIC and HIC contributors, an experienced researcher as chair and was asked to identify 3–5 key research questions. Eleven separate research questions were identified by the three groups (Table 2). The triage question identified 3 separate elements. The outputs from each group were merged to create a long list of research questions to be considered for prioritisation in Phase 4.

**Table 2:** Research questions identified in phase 2

Q1	What are the characteristics of people requiring urgent / emergent care in a particular setting? Groups 1 and 2 including pre-hospital deaths
Q2	What are the obstacles to implementing EC registry / trauma registry-based systems in LMICs? Groups 1, 2 and 3
Q3	How do we describe the journey of a patient through ECS in order to identify barriers to care? Groups 1, 2 and 3. Group 3 includes access differentials imposed by income, geography and discrimination
Q4	Triage: • Where triage systems are existent, what is the accuracy of the triaging system? • Where triage systems do not exist, what are the barriers to implementing triaging systems? • What is the effect of triage on patient outcomes and ECS workload?
Q5	How to develop setting specific, best practice clinical guidelines for emergency care? Group 1
Q6	What is the cost effectiveness of Emergency Care as delivered across the health system (including pre-hospital, emergency unit, inpatient and ICU settings)?Groups 2 and 3
Q7	What are the best quality and access indicators for Emergency Care in LMICs that engage the different stakeholders i.e. community, patients, providers and policy makers? (Groups 2 and 3 also need to measure access of low income groups and return attenders).
Q8	How do you asses the unintended consequences of changing emergency Care systems? Group 2
Q9	What is the impact of pre-hospital care as designed by the WHO ECSA in a country where it previously did not exist? Group 3
Q10	How can countries meet the adequate staffing for Emergency Care delivery including issues of retention, burn out and staff safety? Group 3
Q11	What is the impact of interfacility transfers on cost and effectiveness of the Emergency Care System? Group 3

### Phase 3: situation analysis of research capacity and challenges in low-resource settings

In Phase 3, a situation analysis facilitated reflections on the reality of conducting emergency care research in low-resource settings context. This involved a brief assessment of the current ECS research landscape as compared to the expectations and needs of each country.

Country representatives shared their experiences on the challenges they face in conducting emergency care research in LMICs. Several common themes were identified (Table 3) that could be categorised as factors to do with the external environment such as regulation and policy, the research community present and process issues linked to the conduct of research.

**Table 3:** Challenges of conducting Emergency Care research in LMICs

Themes	Sub-themes
1. External environment	Regulation, policy, local settings, bureaucracy
2. Research community	Brain drain, access to papers, time, collaboration, research capacity
3. Conduct of research	Data collection, data quality, research implementation

## Results

### Phase 4: research prioritisation ranking

In Phase 4, to decide on priorities, a combination of a metrics-based approach (pooling individual rankings), and a consensus-based approach was used. The three groups each ranked the previously highlighted questions according to feasibility and applicability. The roundtable feedback of the scoring from each group enabled a consensus to be reached on the top seven prioritised questions. Of note, between each phase, feedback sessions were conducted.

### The three groups individually scored each of the 11 questions in terms of applicability and feasibility

**Fig. 4:**
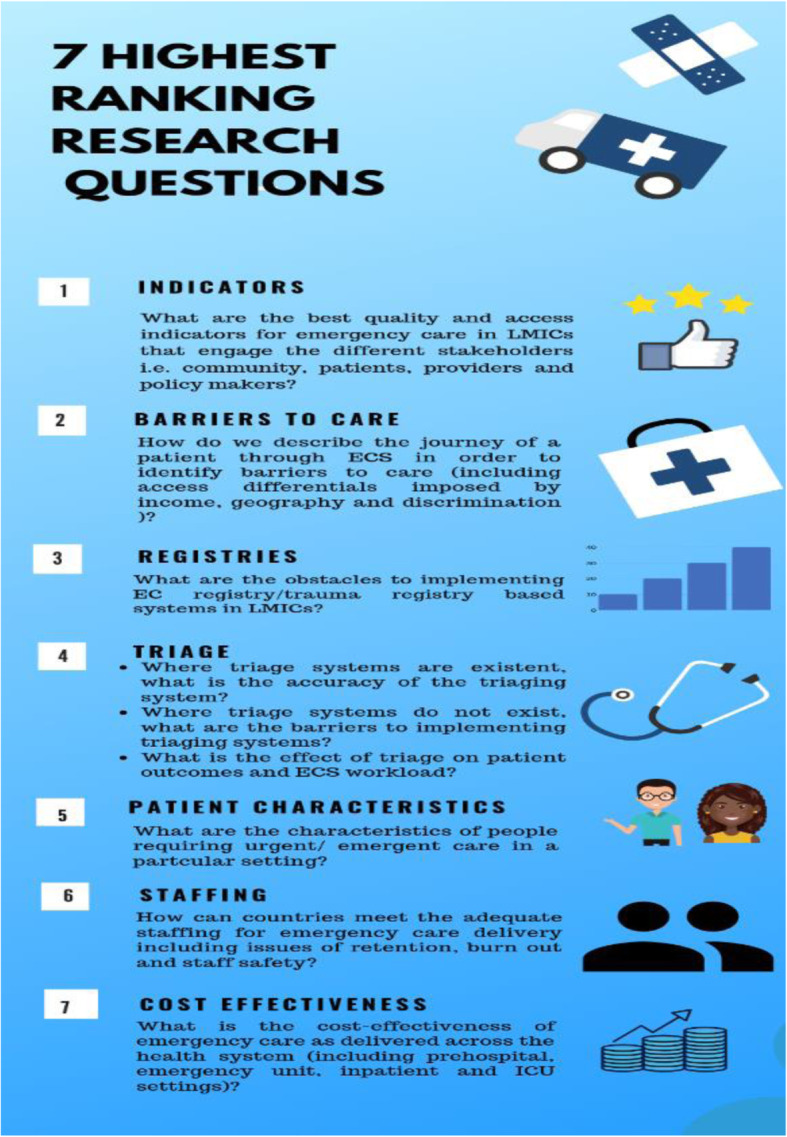
Seven highest ranking Emergency Care research questions in LMICs. Figure created using canva graphic design software https://about.canva.com/license-agreements/free-media/

Merging of the ranking of questions from the 3 groups (appendix 1) identified the top questions. The 7 highest-ranking questions to prioritise (in order of decreasing priority) are listed in Fig. 4:. The top seven priority research questions address identification of context-relevant emergency care indicators, barriers to effective emergency care; accuracy and impact of triage tools; potential quality improvement via registries; characteristics of people seeking emergency care; best practices for staff training and retention; and cost effectiveness of critical care – all within LMICs.

### Quality assessment of the research priority setting exercise

We assessed the quality of the RPS exercise using a checklist of nine themes of good practice as proposed by WHO (Appendix 2) [29]. It adheres to these recommendations considering the context, use of a comprehensive approach, inclusiveness, information gathering, planning for implementation, criteria for deciding on priorities, combination of consensus and metrics based approach and transparency [29]. For example; the focus of the exercise (i.e. what the exercise is about and who it is for) was clearly stated: “to identify and rank important research questions that could improve emergency care in LMICs drawing from cross country experience and expertise.”

Explicit decisions were made as to who to involve (researchers from relevant disciplines and representatives of Ministries of Health in LMICs) in setting the research priorities and why (to enable research priorities to be informed by cross country, multidisciplinary experience and, expertise). This included representation of expertise (researchers from relevant disciplines including Emergency Medicine, Pre-hospital Care, Health Services Research, Public Health, Disaster Management and Defence (Military) Medicine) and regional participation (stakeholders from 13 LMICs and 3 HICs). We deliberately selected high value information to inform the exercise, such as literature reviews, and key guidance documents (as shown in Table 1).

It was communicated to participants that translation of the research priorities to actual research studies could occur via collaborations for global health funding applications and highlighted the importance that research priorities for improving emergency care systems in LMICs are led, developed and informed by local researchers most familiar with the context and working in partnership with their patients. To decide on priorities, we used a combination of a metrics-based approach (pooling individual rankings) informing consensus-based discussions.

We are yet to define when evaluation of the established priorities and the priority setting process will take place but inevitably, the most positive evaluation would result from funding of the prioritised research questions with demonstrable subsequent improvement in corresponding elements of Emergency Care in LMICs.

## Discussion

The multinational, interdisciplinary collaboration supported by the GEMCARN project has conducted research priority setting according to published standards and identified seven research priorities for strengthening Emergency Care in low resource settings - to be taken forward through formal funded studies. These highly-ranked priorities are consistent with the challenges identified in World Health Assembly Resolution 72.16. These include “poor coordination of prehospital and facility-based care; limited or no coverage of prehospital systems, especially in rural areas; shortage of fixed staff assigned to emergency units; lack of standards for clinical management and documentation; and insufficient funding.” [12] Following the RPS exercise, a PubMed search (Oct 03, 2019) was performed using the terms “emergency care”, “research priorit*”, “low income countries”, “middle income countries”, “low-middle income countries”, “developing countries”, “collaboration”, and “network” for articles published in English. The search included technical reports, reviews, books, consensus development conferences, broadly associated with emergency care systems, policies, strategies and data in low- and middle-income countries. We identified supporting evidence in relation to the emergency care research priorities for LMICs:

### Emergency care system indicators

A systematic review of emergency care quality and safety indicators in low resource settings has reported a limited number of metrics, the majority of which focus on structures or processes of care rather than on patient outcomes [30]. A consensus-based set of 76 quality indicators for emergency care in LMICs was produced at the 2016 AFEM, including indicators on mortality outcomes [16]. More recently, International Federation of Emergency Medicine developed a framework for quality and safety, setting out global expectations for emergency care [31]. Therefore, it is worth considering what structure, process and outcome indicators for emergency care reflect the whole patient journey through the ECS in LMICs. The WHO is currently conducting a systematic review on emergency care indicators which will further inform this research priority.

### Barriers to care

A recent systematic review identified six barriers to out-of- hospital care in LMICs [22]. These include culture, infrastructure, communication/coordination, transport, equipment and personnel. However, 56% of the included articles had a primary author from outside of the study country, which means that understanding of the pre-hospital systems maybe limited and the barriers reported may not be the important ones. These barriers are in-line previous research highlighting a lack of coverage of prehospital systems, especially in rural areas, and insufficient coordination among prehospital and facility-based providers [7]. Affordability and a range of socio-economic factors are also key barriers to the ECS as a whole [32].

### Registries

Standardized emergency care registries are largely absent in most LMICs, due to a lack of standard clinical management and documentation in prehospital and facility settings [7, 33]. However, the establishment of registries is slowly increasing in response to the growing body of evidence in support of trauma registries [34, 35]. There are significant challenges to establishing trauma registries in these settings [36]. Barriers to trauma registry implementation include data quality issues, limited resources and, limitations in pre-hospital care. Additional effort is needed to identify effective means of implementation of surveillance and registry systems that are adaptable to different settings including LMICs [33]. A framework for surveillance and registry research in low-resource emergency care settings is clearly needed.

### Triage

Evidence shows that inpatient and emergency department (ED) triage positively predict patient clinical outcomes, safety and waiting times [37–39]. However, there is a dearth of evidence supporting the validity and reliability of triage tools in LMICs [40]. Future research in this field needs to consider changes in research methodology, evaluation of triage tools with actual users, accounting for resource constraints, uniformity in the statistical evaluation and evaluation of triage impact on waiting times, resource utilisation and patient satisfaction [40].

### Patient characteristics

Patients who access emergency care mostly consist of children of median age 3.2 years and adults of median age 35 years [5]. Paediatric patients account for 20–35% of all ED visits globally [41]. These patients have high mortality rates compared to similar patients in high-income countries. Increasingly, a shift is being observed in emergency care surveillance in LMICs to categories of conditions such as non-communicable diseases (NCDs) and injuries [33]. Hence, there is also a need to better describe the disease profile of patients in LMICs who seek emergency care and understand the case mix better, particularly as this will impact on outcomes of these patients, including mortality rates.

### Staffing

Most countries currently face shortages in health care staff but the lack of speciality trained or skilled personnel in emergency care is a particular challenge in LMICs [42–46]. It has been recommended that basic lifesaving skills and first-aid training is needed for pre-hospital providers, taxi drivers and the police especially in settings where emergency responders do not exist [22]. There are also other staffing issues such as rotation of staff and security issues for staff in the emergency department that impact emergency care services.

### Cost effectiveness

In the context of resource constraints common to virtually all health system settings, the cost effectiveness of interventions and services delivered is paramount. The World Bank’s Disease Control Priorities in Developing Countries has identified the most effective and cost-effective interventions across a wide range of disease conditions [47]. Some examples of highly cost-effective emergency care services in LMICs have also been identified including the provision of a “dedicated emergency unit with formal triage, oxygen for pneumonia, pulse oximetry for childhood pneumonia, treatment of acute myocardial infarction, emergency obstetric care, trauma surgery and emergency obstetric services” [7]. However, studies of the cost effectiveness of emergency care in LMICs are still at an early stage and there is a dearth of high quality evidence. This includes for example common approaches such as task-shifting from doctors to allied health professionals such as community health workers and nurses or from health facility into the community that are likely to be cost-effective and even potentially cost-saving but for which there is little strong evidence.

## Conclusion

Ultimately, an evidence-to-policy stance in emergency care will be crucial for effective development in LMICs. Despite the highlighted challenging factors of external environment, research community and conduct of research facing emergency care research in LMICs, this RPS exercise has identified key research priorities to support the development of evidence, research capacity and to inform efforts to improve ECS in low-resource settings. This can guide future research and funding applications to support emergency care development for the world’s poorest billion. Such collaborations as these draw on the strength of the “South-South” cross-learning among LMIC partners in addition to a mutual reinforcement between high-income and LMIC collaborators, allowing for HIC collaborators alike to learn from the relative effectiveness of various emergency care interventions as they seek to further understand and strengthen ECS. Gradually, we will be able to build research capacity such that interventions to improve emergency care systems in LMICs will be led, developed and informed by local researchers most familiar with the context.

## Supplementary information

**Additional file 1.**

## Data Availability

All source data is provided within the manuscript, the corresponding author can address further questions.
