# Supplementary material for: Harnessing inter-disciplinary collaboration to improve emergency care in low- and middle-income countries (LMICs): results of research prioritisation setting exercise
Source: BMC Emerg Med. 2020 Aug 31;20:68. doi: 10.1186/s12873-020-00362-7 (PMC7457362; doi:10.1186/s12873-020-00362-7)
Supplement: Supplementary file 1 — Additional file 1. [file 12873_2020_362_MOESM1_ESM.docx]

APPENDIX 1: Ranking of Research questions in Groups 1, 2 & 3 and merged ranking

|  | **GROUP 1** | **GROUP 2** | **GROUP 3** | **TOTAL** |
| --- | --- | --- | --- | --- |
| Q1. | 5 | 3/1 | 7 | 13/15 |
| Q2. | 2 | 5 | 7 | 14 |
| Q3. | 3 | 4 | 3 | 10 |
| Q4. | 1 | 8 | 5 | 14 |
| Q5. | 11 | 9 | 10 | 30 |
| Q6. | 6 | 6 | 5 | 17 |
| Q7. | 4 | 2 | 2 | **8** |
| Q8. | 10 | 11 | 11 | 32 |
| Q9. | 8 | 9 | 3 | 20 |
| Q10. | 9 | 7 | 1 | 17 |
| Q11. | 6 | 12 | 7 | 25 |

|  |  | **GROUP 1** | **GROUP 2** | **GROUP 3** | **TOTAL** |
| --- | --- | --- | --- | --- | --- |
| Q1 | What are the characteristics of people requiring urgent / emergent care in a particular setting? Groups 1 and 2 including pre-hospital deaths | 5 | 3/1 | 7 | 13/15 |
| Q2 | What are the obstacles to implementing EC registry / trauma registry-based systems in LMICs? Groups 1, 2 and 3 | 2 | 5 | 7 | 14 |
| Q3 | How do we describe the journey of a patient through ECS in order to identify barriers to care? Groups 1, 2 and 3. Group 3 includes access differentials imposed by income, geography and discrimination | 3 | 4 | 3 | 10 |
| Q4 | Triage:   - Where triage systems are existent, what is the accuracy of the triaging system? - Where triage systems do not exist, what are the barriers to implementing triaging systems? - What is the effect of triage on patient outcomes and ECS workload? | 1 | 8 | 5 | 14 |
| Q5 | How to develop setting specific, best practice clinical guidelines for emergency care? Group 1 | 11 | 9 | 10 | 30 |
| Q6 | What is the cost effectiveness of Emergency Care as delivered across the health system (including pre-hospital, emergency unit, inpatient and ICU settings)?Groups 2 and 3 | 6 | 6 | 5 | 17 |
| Q7 | What are the best quality and access indicators for Emergency Care in LMICs that engage the different stakeholders i.e. community, patients, providers and policy makers?(Groups 2 and 3 also need to measure access of low income groups and return attenders). | 4 | 2 | 2 | **8** |
| Q8 | How do you asses the unintended consequences of changing emergency Care systems? Group 2 | 10 | 11 | 11 | 32 |
| Q9 | What is the impact of pre-hospital care as designed by the WHO ECSA in a country where it previously did not exist? Group 3 | 8 | 9 | 3 | 20 |
| Q10 | How can countries meet the adequate staffing for Emergency Care delivery including issues of retention, burn out and staff safety?Group 3 | 9 | 7 | 1 | 17 |
| Q11 | What is the impact of interfacility transfers on cost and effectiveness of the Emergency Care System? Group 3 | 6 | 12 | 7 | 25 |

APPENDIX 2: **Checklist for health research priority setting**

| **Preparatory work** |
| --- |
| **1. Context** |
| Decide which contextual factors underpin the process: What resources are available for the exercise? What is the focus of the exercise (i.e. what is the exercise about and who is it for)? What are the underlying values or principles? What is the health, research and political environment in which the process will take place? |
| **2. Use of a comprehensive approach** |
| Decide if use of a comprehensive approach is appropriate, or if development of own methods is the preferred choice. These approaches provide structured, detailed, step-by-step guidance for health research priority setting processes from beginning to end. |
| **3. Inclusiveness** |
| Decide who should be involved in setting the health research priorities and why. Is there appropriate representation of expertises and balanced gender and regional participation? Have important health sectors and other constituencies been included? |
| **4. Information gathering** |
| Choose what information should be gathered to inform the exercise, such as literature reviews, collection of technical data (e.g. burden of disease or cost-effectiveness data), assessment of broader stakeholder views, reviews or impact analyses of previous priority setting exercises or exercises from other geographical levels. |
| **5. Planning for implementation** |
| Establish plans for translation of the priorities to actual research (via policies and funding) as a priority at the beginning of the process. Who will implement the research priorities? And how? |
| **Deciding on priorities** |
| **6. Criteria** |
| Select relevant criteria to focus discussion around setting priorities. |
| **7. Methods for deciding on priorities** |
| Choose a method for deciding on priorities. Decide whether to use a consensus based approach or a metrics based approach (pooling individual rankings), or a combination. |
| **After priorities have been set** |
| **8. Evaluation** |
| Define when and how evaluation of the established priorities and the priority setting process will take place. Health research priority setting should not be a one-time exercise! |
| **9. Transparency** |
| Write a clear report that discusses the approach used: Who set the priorities? How exactly were the priorities set? |
